# Synthesis, *in vitro* screening and molecular docking of isoquinolinium-5-carbaldoximes as acetylcholinesterase and butyrylcholinesterase reactivators

**DOI:** 10.1080/14756366.2019.1710501

**Published:** 2020-01-07

**Authors:** David Malinak, Rafael Dolezal, Vendula Hepnarova, Miroslava Hozova, Rudolf Andrys, Petr Bzonek, Veronika Racakova, Jan Korabecny, Lukas Gorecki, Eva Mezeiova, Miroslav Psotka, Daniel Jun, Kamil Kuca, Kamil Musilek

**Affiliations:** a Department of Chemistry, Faculty of Science, University of Hradec Kralove, Hradec Kralove, Czech Republic; b Biomedical Research Centre, University Hospital in Hradec Kralove, Hradec Kralove, Czech Republic; c Department of Toxicology and Military Pharmacy, Faculty of Military Health Sciences, University of Defence, Hradec Kralove, Czech Republic; d Faculty of Informatics and Management, Center for Basic and Applied Research, University of Hradec Kralove, Hradec Kralove, Czech Republic

**Keywords:** Acetylcholinesterase, butyrylcholinesterase, organophosphate, reactivator, oxime

## Abstract

The series of symmetrical and unsymmetrical isoquinolinium-5-carbaldoximes was designed and prepared for cholinesterase reactivation purposes. The novel compounds were evaluated for intrinsic acetylcholinesterase (AChE) or butyrylcholinesterase (BChE) inhibition, when the majority of novel compounds resulted with high inhibition of both enzymes and only weak inhibitors were selected for reactivation experiments on human AChE or BChE inhibited by sarin, VX, or paraoxon. The AChE reactivation for all used organophosphates was found negligible if compared to the reactivation ability of obidoxime. Importantly, two compounds were found to reactivate BChE inhibited by sarin or VX better to obidoxime at human attainable concentration. One compound resulted as better reactivator of NEMP (VX surrogate)-inhibited BChE than obidoxime. The *in vitro* results were further rationalized by molecular docking studies showing future directions on designing potent BChE reactivators.

## GRAPHICAL ABSTRACT

**Figure:**
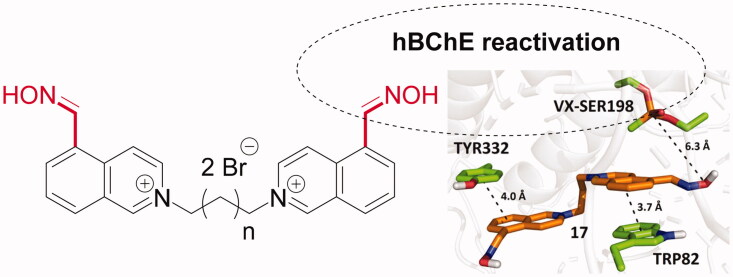

## Introduction

**Figure 1.**
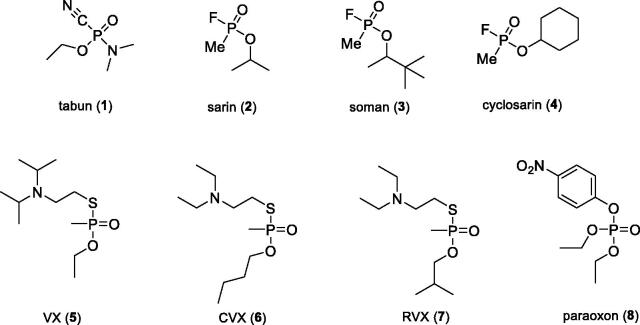
The structures of G- and V-nerve agent series and pesticide paraoxon.

The function of the human nervous system is based on a complex interaction of nerves through neurotransmitters that maintain the activity of organs, glands, and other neurons in dynamic equilibrium^1^^,^^2^. This is done with the help of chemical synapses where the neurotransmitter released from the presynaptic terminus of the axon diffuses through a narrow synaptic cleft and postsynaptically binds to the respective receptors of the subsynaptic membrane of the neuron, gland, or muscle cells^1^. These very small and important molecules include, for example, acetylcholine (ACh), dopamine, histamine, serotonin etc. The processing of ACh may be seriously compromised when ACh receptors are blocked or the hydrolytic activity of acetylcholinesterase (AChE; EC 3.1.1.7) is impaired. The degradation of ACh is thus not proceeding and neurotransmitter overstimulates cholinergic receptors^2^^,^^3^. The most known irreversible AChE inhibitors are organophosphates (OPs), which belong to the most dangerous and lethal substances developed by man^4^^,^^5^. OPs are a heterogeneous group of organic compounds that are a serious toxicological problem. These synthetic compounds have been discovered since the first half of 20th century^6^. To date, they were misused in several military conflicts or terrorist attacks. They are also extensively used in agriculture as insecticides^4^^,^^7^. Nerve agents can be divided into two groups. The first group labelled “G-series” includes e.g. tabun (**1**), sarin (**2**), soman (**3**), and cyclosarin (**4**) (Figure 1). These agents are volatile, therefore, their solutions and vapours are very dangerous, even though their persistence in open terrain is low. The second group, referred to as the “V-series,” contains e.g. VX (**5**), CVX - the Chinese isomer (**6**) and RVX - the Russian isomer (**7**) (Figure 1)^5^. This group of agents is relatively non-volatile, has high persistence in the environment and enhanced toxicity^8^.

**Figure 2.**
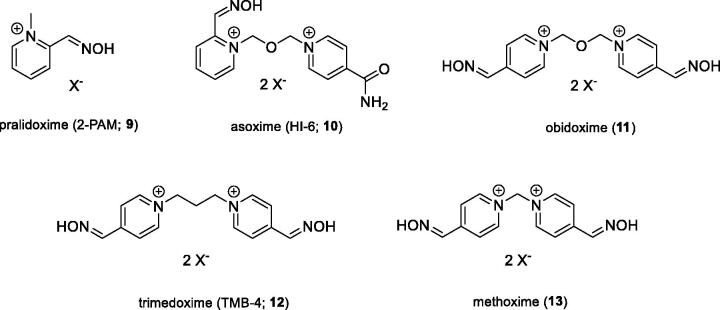
Structures of AChE reactivators.

The second large group of OPs are insecticides, including e.g. chlorpyriphos, paraoxon (**8**), or methyl paraoxon, which might pose a threat to civilians due to their extensive agricultural use^9^. The pesticides annually cause about 3 million of acute intoxications, with over 260,000 of them end fatally^6^. Some of these cases are deliberate suicides^4^. The main toxic mechanism of the OPs is based on the irreversible inhibition of the AChE enzyme, whose main biological function is the hydrolysis of the positively charged neurotransmitter ACh^8^. Inhibition thus leads to accumulation of ACh in cholinergic synapses and excessive stimulation of cholinergic receptors of muscarinic and nicotinic type. This pathological condition is referred to as “cholinergic crisis,” which is characterised by: sweating, saliva, diarrhoea, tremors, muscle twitching, or increased gastrointestinal effects. Death is caused by respiratory failure^6^. The current antidotal treatment of OPs poisoning involves the administration of an anticholinergic agent atropine that blocks excessive stimulation of muscarinic receptors, and oximes that are capable of reactivating irreversibly inhibited AChE. The reactivators as pralidoxime (**9**), asoxime (syn. HI-6; **10**), obidoxime (**11**), trimedoxime (**12**), and methoxime (**13**) (Figure 2) are approved as an antidotes against nerve agents, but unfortunately, their efficacy in suppressing acute toxic effects of all types of nerve agents is rather limited^10^. However, these are the most studied and commercially available reactivators^8^.

In this work, the novel isoquinoline-5-carbaldoximes were designed, prepared and evaluated for the reactivation properties of phosphylated human acetylcholinesterase (*h*AChE) and butyrylcholinesterase (*h*BChE).

## Experimental

### Chemistry

All commercial reagents and solvents used were of the highest available purity from Sigma-Aldrich (Prague, Czech Republic). For flash column chromatography on silica gel, Kieselgel 60 (0.063–0.200 mm, 70–230 mesh, Fluka) was used. Solvents for flash column chromatography were purchased from Penta Chemicals Unlimited (Prague, Czech Republic). Thin-layer chromatography was run on Merck silica gel 60 F_254_ analytical plates; detection was carried out with ultraviolet light (254 nm). Melting points were recorded on a Melting Point Apparatus – Stuart SMP30 and are uncorrected. The ^1^H NMR and ^13^C NMR spectra were recorded in DMSO-d_6_ and CD_3_OD-d_4_ solution at ambient temperature on a Varian S500 spectrometer (499.87 MHz for ^1^H and 125.71 MHz for ^13^C). Chemical shifts, *δ*, are given in parts per million (ppm), and spin multiplicities are given as s (singlet), br s (broad singlet), d (doublet), t (triplet), or m (multiplet). Coupling constants, *J*, are expressed in hertz (Hz). For ^1^H *δ* relative to DMSO-d_6_ (*δ* = 2.50) or CD_3_OD-d_4_ (*δ* = 3.31) and for ^13^C relative to DMSO-d_6_ (*δ* = 39.43) or CD_3_OD-d_4_ (*δ* = 49.05). High Resolution Mass Spectrometry (HRMS) was determined by Q Exactive Plus hybrid quadrupole-orbitrap spectrometer.

### *Synthesis of isoquinoline-5-carbaldehyde oxime (*15*)*

To a solution of isoquinoline-5-carboxaldehyde (**14**) (5.00 g, 31.81 mM) in EtOH (500 ml) was added the 50% aq. sol. NH_2_OH (3.90 ml, 63.63mM). The resulting mixture was stirred for 24 h at room temperature. Subsequently, the mixture was concentrated under vacuum and the residue was chromatographed on silica gel (heptane-EtOAc, 1:1) to afford 3.85 g (70%) of compound **15** as a white solid, mp: 158.3–160.3 °C. ^1^H NMR (500 MHz, DMSO-d_6_): *δ* 7.69–7.72 (m, 1H, ArH), 8.03 (d, *J*= 7.2 Hz, 1H, ArH), 8.14 (d, *J=* 8.1 Hz, 1H, ArH), 8.52–8.58 (m, 2H, 2 × ArH), 8.76 (s, 1H, CH), 9.35 (s, 1H, ArH), 11.62 (s, 1H, OH). ^13^C NMR (126 MHz, DMSO-d_6_): *δ* 117.6, 127.0, 127.9, 128.5, 129.2, 130.7, 132.2, 143.8, 147.5, and 152.9. HRMS (HESI^+^): [M + H]^+^: calculated for C_10_H_9_N_2_O^+^ (*m/z*): 173.0709; found: 173.0707.

### Synthesis of 5-((hydroxyimino)methyl)-2-methylisoquinolin-2-ium iodide (16)

Methyl iodide (CH_3_I) (0.36 ml, 5.80 mM) was added at room temperature to isoquinoline-5-carbaldehyde oxime (**15**) (500 mg, 2.90 mM) in EtOH (5 ml), and the resulting mixture was stirred under reflux for 24 h. Subsequently hot mixture was filtered, washed with hot EtOH and allowed to dry at room temperature. The compound **16** was isolated as a yellow solid in yield 650 mg (71%), mp: 220.0–221.5 °C. ^1^H NMR (500 MHz, DMSO-d_6_): *δ* 4.49 (s, 3H, CH_3_), 8.07–8.10 (m, 1H, ArH), 8.45–8.49 (m, 2H, 2 × ArH), 8.79 (d, *J*= 7.1 Hz, 1H, ArH), 8.88 (s, 1H, CH), 9.12 (d, *J*= 7.1 Hz, 1H, ArH), 10.08 (s, 1H, ArH), 11.94 (s, 1H, OH). ^13^C NMR (126 MHz, DMSO-d_6_): *δ* 47.7, 122.7, 127.7, 128.9, 130.8, 131.2, 133.5, 135.7, 136.4, 146.4, and 151.0. HRMS (HESI^+^): [M + H]^+^: calculated for C_11_H_11_N_2_O^+^ (*m/z*): 187.0866; found: 187.0863.

### Synthesis of bisisoquinolinium salts 17–26

To a solution of isoquinoline-5-carbaldehyde oxime (**15**) (100 mg, 0.58 mM) in dimethylformamide (DMF; 0.5 ml) was added the dibromoalkane (0.26 mM). The resulting mixture was stirred for 48 h at 73 °C. The solvent was evaporated under reduced pressure, and the crude product purified by crystallisation from acetone, filtered, washed with acetone and allowed to dry at room temperature.

### 2,2′-(Propane-1,3-diyl)bis(5-((hydroxyimino)methyl)isoquinolin-2-ium) bromide (17)

Compound **17** was isolated as white solid, yield 100 mg (70%), mp: 249.0–251.0 °C. ^1^H NMR (500 MHz, DMSO-d_6_): *δ* 2.93–2.98 (m, 2H, CH_2_), 4.98 (t, *J*= 7.0 Hz, 4H, 2 × CH_2_), 8.09–8.12 (m, 2H, 2 × ArH), 8.47–8.52 (m, 4H, 4 × ArH), 8.89 (s, 2H, 2 × CH), 9.01 (d, *J*= 7.0 Hz, 2H, 2 × ArH), 9.17 (d, *J*= 7.1 Hz, 2H, 2 × ArH), 10.35 (s, 2H, 2 ×ArH), 11.96 (s, 2H, 2 × OH). ^13^C NMR (126 MHz, DMSO-d_6_): *δ* 30.9, 57.3, 123.3, 128.0, 129.0, 130.9, 131.5, 133.9, 135.5, 136.0, 146.3, and 150.8. HRMS (HESI^+^): [M]^2+^: calculated for C_23_H_22_N_4_O_2_^2+^ (*m/z*): 193.0866; found: 193.0865.

### 2,2′-(Butane-1,4-diyl)bis(5-((hydroxyimino)methyl)isoquinolin-2-ium) bromide (18)

Compound **18** was isolated as yellow solid, yield 124 mg (85%), mp: 246.5–248.5 °C. ^1^H NMR (500 MHz, CD_3_OD-d_4_): *δ* 2.27–2.33 (m, 4H, 2 × CH_2_), 4.89–4.92 (m, 4H, 2 × CH_2_), 8.05–8.10 (m, 2H, 2 ×ArH), 8.39–8.47 (m, 4H, 4 × ArH), 8.72–8.79 (m, 4H, 2 × ArH and 2 × CH), 9.36–9.39 (m, 2H, 2× ArH), 9.97–10.04 (m, 2H, 2 × ArH). HRMS (HESI^+^): [M]^2+^: calculated for C_24_H_24_N_4_O_2_^2+^ (*m/z*): 200.0944; found: 200.0942.

### *2,2′-(Pentane-1,5-diyl)bis(5-((hydroxyimino)methyl)isoquinolin-2-ium) bromide (*19*)*

Compound **19** was isolated as brown solid, yield 140 mg (94%), mp: 207.0–209.0 °C. ^1^H NMR (500 MHz, CD_3_OD-d_4_): *δ* 1.58–1.65 (m, 2H, CH_2_), 2.25–2.31 (m, 4H, 2 × CH_2_), 4.84 (t, *J*= 7.5 Hz, 4H, 2 × CH_2_), 8.05–8.08 (m, 2H, 2 × ArH), 8.38 (d, *J* = 7.2 Hz, 2H, 2 × ArH), 8.48 (d, *J*= 8.3 Hz, 2H, 2 × ArH), 8.71 (s, 2H, 2 × CH), 8.75 (d, *J*= 7.1 Hz, 2H, 2 × ArH), 9.33 (d, *J*= 7.0 Hz, 2H, 2 × ArH), 10.04 (s, 2H, 2 × ArH). ^13^C NMR (126 MHz, CD_3_OD-d_4_): *δ* 23.9, 31.5, 62.3, 125.9, 130.2, 131.3, 132.4, 132.6, 136.2, 136.4, 138.7, 148.2, and 151.3. HRMS (HESI^+^): [M]^2+^: calculated for C_25_H_26_N_4_O_2_^2+^ (*m/z*): 207.1022; found: 207.1020.

### *2,2′-(Hexane-1,6-diyl)bis(5-((hydroxyimino)methyl)isoquinolin-2-ium) bromide (*20*)*

Compound **20** was isolated as white solid, yield 69 mg (45%), mp: 249.5–250.5 °C. ^1^H NMR (500 MHz, CD_3_OD-d_4_): *δ* 1.57–1.60 (m, 4H, 2 × CH_2_), 2.15–2.21 (m, 4H, 2 × CH_2_), 4.79–4.82 (m, 4H, 2 × CH_2_), 8.06–8.09 (m, 2H, 2 × ArH), 8.40 (d, *J*= 7.2 Hz, 2H, 2 × ArH), 8.47 (d, *J*= 8.3 Hz, 2H, 2 ×ArH), 8.73–8.76 (m, 4H, 2 × ArH and 2 × CH), 9.37 (d, *J*= 7.1 Hz, 2H, 2 × ArH), 10.02 (s, 2H, 2 × ArH). HRMS (HESI^+^): [M]^2+^: calculated for C_26_H_28_N_4_O_2_^2+^ (*m/z*): 214.1101; found: 214.1098.

### *2,2′-(Heptane-1,7-diyl)bis(5-((hydroxyimino)methyl)isoquinolin-2-ium) bromide (*21*)*

Compound **21** was isolated as white solid, yield 140 mg (89%), mp: 242.0–243.5 °C. ^1^H NMR (500 MHz, CD_3_OD-d_4_): *δ* 1.47–1.56 (m, 6H, 3 × CH_2_), 2.13–2.19 (m, 4H, 2 × CH_2_), 4.78–4.81 (m, 4H, 2 × CH_2_), 8.06–8.09 (m, 2H, 2 × ArH), 8.39 (d, *J*= 7.3 Hz, 2H, 2 × ArH), 8.47 (d, *J*= 8.4 Hz, 2H, 2 × ArH), 8.72 (s, 2H, 2 ×CH), 8.76 (d, *J*= 7.1 Hz, 2H, 2 × ArH), 9.36 (d, *J*= 7.1 Hz, 2H, 2 × ArH), 10.02 (s, 2H, 2× ArH). ^13^C NMR (126 MHz, CD_3_OD-d_4_): *δ* 27.0, 29.5, 32.1, 62.7, 125.9, 130.2, 131.3, 132.4, 132.5, 136.1, 136.4, 138.6, 148.2, and 151.3. HRMS (HESI^+^): [M]^2+^: calculated for C_27_H_30_N_4_O_2_^2+^ (*m/z*): 221.1179; found: 221.1177.

### *2,2′-(Octane-1,8-diyl)bis(5-((hydroxyimino)methyl)isoquinolin-2-ium) bromide (*22*)*

Compound **22** was isolated as white solid, yield 71 mg (44%), mp: 246.0–247.1 °C. ^1^H NMR (500 MHz, CD_3_OD-d_4_): *δ* 1.44–1.49 (m, 8H, 4 × CH_2_), 2.11–2.18 (m, 4H, 2 ×CH_2_), 4.76–4.79 (m, 4H, 2 × CH_2_), 8.06–8.09 (m, 2H, 2 × ArH), 8.40 (d, *J*= 7.3 Hz, 2H, 2 × ArH), 8.47 (d, *J*= 8.2 Hz, 2H, 2 × ArH), 8.73–8.76 (m, 4H, 2 × ArH and 2 × CH), 9.37 (d, *J*= 7.1 Hz, 2H, 2 × ArH), 10.00 (s, 2H, 2 × ArH). HRMS (HESI^+^): [M]^2+^: calculated for C_28_H_32_N_4_O_2_^2+^ (*m/z*): 228.1257; found: 228.1255.

### *2,2′-(Nonane-1,9-diyl)bis(5-((hydroxyimino)methyl)isoquinolin-2-ium) bromide (*23*)*

Compound **23** was isolated as white solid, yield 128 mg (78%), mp: 247.5–248.5 °C. ^1^H NMR (500MHz, CD_3_OD-d_4_): *δ* 1.40–1.50 (m, 10H, 5 × CH_2_), 2.11–2.17 (m, 4H, 2 × CH_2_), 4.78 (t, *J*= 7.6 Hz, 4H, 2 × CH_2_), 8.06–8.09 (m, 2H, 2 × ArH), 8.39 (d, *J*= 7.3 Hz, 2H, 2 × ArH), 8.47 (d, *J*= 8.3 Hz, 2H, 2 × ArH), 8.72 (s, 2H, 2 × CH), 8.75 (d, *J*= 6.9 Hz, 2H, 2 × ArH), 9.36 (d, *J*= 7.0 Hz, 2H, 2 × ArH), 10.01 (s, 2H, 2 × ArH). ^13^C NMR (126 MHz, CD_3_OD-d_4_): *δ* 27.3, 30.0, 30.2, 32.3, 62.9, 125.8, 130.2, 131.2, 132.4, 132.5, 136.1, 136.4, 138.6, 148.2, and 151.2. HRMS (HESI^+^): [M]^2+^: calculated for C_29_H_34_N_4_O_2_^2+^ (*m/z*): 235.1335; found: 235.1333.

### *2,2′-(Decane-1,10-diyl)bis(5-((hydroxyimino)methyl)isoquinolin-2-ium) bromide (*24*)*

Compound **24** was isolated as white solid, yield 84 mg (50%), mp: 240.5–241.5 °C. ^1^H NMR (500 MHz, DMSO-d_6_): *δ* 1.25–1.31 (m, 12H, 6 × CH_2_), 1.98–2.07 (m, 2H, 2 × CH_2_), 4.74 (t, *J*= 6.8 Hz, 4H, 2 × CH_2_), 8.08–8.12 (m, 2H, 2 × ArH), 8.46–8.52 (m, 4H, 4 × ArH), 8.89 (s, 2H, 2 × CH), 8.95 (d, *J*= 6.7 Hz, 2H, 2 × ArH), 9.16 (d, *J*= 6.8 Hz, 2H, 2 × ArH), 10.27 (s, 2H, 2 × ArH), 11.94 (s, 2H, 2 × OH). ^13^C NMR (126 MHz, DMSO-d_6_): *δ* 25.4, 28.3, 28.6, 30.3, 60.5, 123.2, 127.9, 129.0, 130.8, 131.4, 133.8, 135.5, 135.8, 146.4, and 150.3. HRMS (HESI^+^): [M]^2+^: calculated for C_30_H_36_N_4_O_2_^2+^ (*m/z*): 242.1414; found: 242.1409.

### *2,2′-(Undecane-1,11-diyl)bis(5-((hydroxyimino)methyl)isoquinolin-2-ium) bromide (*25*)*

Compound **25** was isolated as yellow oil, yield 102 mg (60%). ^1^H NMR (500 MHz, CD_3_OD-d_4_): *δ* 1.28–1.48 (m, 14H, 7 × CH_2_), 2.11–2.17 (m, 4H, 2 × CH_2_), 4.78–4.81 (m, 4H, 2 × CH_2_), 8.04–8.08 (m, 2H, 2 × ArH), 8.37 (d, *J*= 7.2 Hz, 2H, 2 × ArH), 8.48 (d, *J*= 8.3 Hz, 2H, 2 × ArH), 8.70 (s, 2H, 2 × CH), 8.77 (d, *J*= 7.1 Hz, 2H, 2× ArH), 9.34 (d, *J*= 7.1 Hz, 2H, 2 × ArH), 10.04 (s, 2H, 2 × ArH). ^13 ^C NMR (126 MHz, CD_3_OD-d_4_): *δ* 27.3, 30.1, 30.4, 30.4, 32.3, 62.9, 125.8, 130.2, 131.1, 132.3, 132.5, 136.0, 136.5, 138.5, 148.2, and 151.2. HRMS (HESI^+^): [M]^2+^: calculated for C_31_H_38_N_4_O_2_^2+^ (*m/z*): 249.1492; found: 249.1488.

### *2,2′-(Dodecane-1,12-diyl)bis(5-((hydroxyimino)methyl)isoquinolin-2-ium) bromide (*26*)*

Compound **26** was isolated as yellow solid, yield 58 mg (33%), mp: 70.5–72.5 °C. ^1^H NMR (500 MHz, CD_3_OD-d_4_): *δ* 1.27–1.48 (m, 16H, 8 ×CH_2_), 2.10–2.16 (m, 4H, 2 × CH_2_), 4.79 (t, *J*= 7.6 Hz, 4H, 2 ×CH_2_), 8.05–8.08 (m, 2H, 2 × ArH), 8.37 (d, *J*= 7.3 Hz, 2H, 2× ArH), 8.48 (d, *J*= 8.2 Hz, 2H, 2 × ArH), 8.70 (s, 2H, 2 × CH), 8.76 (d, *J*= 7.1 Hz, 2H, 2 × ArH), 9.35 (d, *J*= 7.1 Hz, 2H, 2 × ArH), 10.03 (s, 2H, 2 × ArH). ^13^C NMR (126 MHz, CD_3_OD-d_4_): *δ* 27.3, 30.1, 30.5, 30.6, 32.4, 62.9, 125.8, 130.2, 131.2, 132.3, 132.5, 136.0, 136.5, 138.5, 148.2, and 151.2. HRMS (HESI^+^): [M]^2+^: calculated for C_32_H_40_N_4_O_2_^2+^ (*m/z*): 256.1570; found: 256.1567.

### Synthesis of unsymmetrical isoquinolinium-pyridiniumamide bisquaternary salts 30–32

To a solution of monoquaternary isonicotinamide salts **27**–**29** (0.45 mM) in DMF (0.5 ml) was added isoquinoline-5-carbaldehyde oxime (**15**) (0.58 mM). The resulting mixture was stirred for 48 h at 73 °C. The solvent was evaporated under reduced pressure, and the crude product purified by crystallisation from EtOH, filtered, washed with EtOH and allowed to dry at room temperature.

### *2–(3-(4-carbamoylpyridinium-1-yl)propyl)-5-((hydroxyimino)methyl)isoquinolin-2-ium bromide (*30*)*

Compound **30** was isolated as white solid, yield 171 mg (77%), mp: 261.0–262.5 °C. ^1^H NMR (500 MHz, DMSO-d_6_): *δ* 2.81–2.87 (m, 2H, CH_2_), 4.88–4.94 (m, 4H, 2 × CH_2_), 8.10–8.14 (m, 1H, ArH), 8.30 (br s, 1H, NH), 8.49–8.55 (m, 4H, 4× ArH), 8.74 (br s, 1H, NH), 8.91 (s, 1H, CH), 9.00 (d, *J*= 7.1 Hz, 1H, ArH), 9.20 (d, *J*= 7.1 Hz, 1H, ArH), 9.38 (d, *J*= 6.8 Hz, 2H, 2 × ArH), 10.34 (s, 1H, ArH), 11.95 (s, 1H, OH). ^13^C NMR (126 MHz, DMSO-d_6_): *δ* 31.3, 57.1, 57.4, 123.3, 125.9, 128.0, 129.0, 130.9, 131.5, 134.0, 135.6, 136.0, 145.9, 146.4, 148.1, 150.8, and 163.1. HRMS (HESI^+^): [M]^2+^: calculated for C_19_H_20_N_4_O_2_^2+^ (*m/z*): 168.0788; found: 168.0785.

### *2–(4-(4-carbamoylpyridinium-1-yl)butyl)-5-((hydroxyimino)methyl)isoquinolin-2-ium bromide (*31*)*

Compound **31** was isolated as white solid, yield 122 mg (53%), mp: 138.5–140.0 °C. ^1^H NMR (500 MHz, DMSO-d_6_): *δ* 2.05–2.14 (m, 4H, 2 × CH_2_), 4.77–4.85 (m, 4H, 2 × CH_2_), 8.09–8.12 (m, 1H, ArH), 8.28 (br s, 1H, NH), 8.46–8.48 (m, 3H, 3 × ArH), 8.52 (d, *J*= 8.2 Hz, 1H, ArH), 8.71 (br s, 1H, NH), 8.89 (s, 1H, CH), 8.97 (d, *J*= 7.1Hz, 1H, ArH), 9.17 (d, *J*= 7.1 Hz, 1H, ArH), 9.34 (d, *J*= 6.5 Hz, 2H, 2 × ArH), 10.32 (s, 1H, ArH), 11.95 (s, 1H, OH). ^13^C NMR (126 MHz, DMSO-d_6_): *δ* 26.7, 27.0, 59.6, 59.8, 123.2, 125.8, 128.0, 129.0, 130.8, 131.5, 133.9, 135.5, 135.9, 145.7, 146.4, 148.0, 150.5, and 163.2. HRMS (HESI^+^): [M]^2+^: calculated for C_20_H_22_N_4_O_2_^2+^ (*m/z*): 175.0866; found: 175.0865.

### *2–(5-(4-carbamoylpyridinium-1-yl)pentyl)-5-((hydroxyimino)methyl)isoquinolin-2-ium bromide (*32*)*

Compound **32** was isolated as yellow solid, yield 164 mg (70%), mp: 232.5–233.5 °C. ^1^H NMR (500 MHz, DMSO-d_6_): *δ* 1.35–1.41 (m, 2H, CH_2_), 2.01–2.15 (m, 4H, 2 × CH_2_), 4.70–4.79 (m, 4H, 2 × CH_2_), 8.09–8.13 (m, 1H, ArH), 8.29 (br s, 1H, NH), 8.47–8.48 (m, 3H, 3 × ArH), 8.54 (d, *J*= 8.3 Hz, 1H, ArH), 8.72 (br s, 1H, NH), 8.90 (s, 1H, CH), 8.99 (d, *J*= 7.1 Hz, 1H, ArH), 9.18 (d, *J*= 7.1 Hz, 1H, ArH), 9.37 (d, *J*= 6.7 Hz, 2H, 2 × ArH), 10.35 (s, 1H, ArH), 11.95 (s, 1H, OH). ^13^C NMR (126 MHz, DMSO-d_6_): *δ* 21.8, 29.4, 29.8, 59.9, 60.2, 123.2, 125.7, 127.9, 129.0, 130.8, 131.4, 133.8, 135.6, 135.9, 145.7, 146.4, 148.0, 150.4, and 163.2. HRMS (HESI^+^): [M]^2+^: calculated for C_21_H_24_N_4_O_2_^2+^ (*m/z*): 182.0944; found: 182.0942.

### *Synthesis of 2,2′-(oxybis(methylene))bis(5-((hydroxyimino)methyl)isoquinolin-2-ium) chloride (*33*)*

Bis(chloromethyl)ether (0.025 ml, 0.29 mM) was added at room temperature to isoquinoline-5-carbaldehyde oxime (15) (100 mg, 0.58 mM) in DMF (0.5 ml), and the resulting mixture was stirred for 48 h at 73 °C. The solvent was evaporated under reduced pressure, and the crude product purified by crystallisation from EtOH, filtered, washed with EtOH and allowed to dry at room temperature. The compound **33** was isolated as a yellow solid in yield 60 mg (45%), mp: 182.5–184.5 °C. ^1^H NMR (500 MHz, CD_3_OD-d_4_): *δ* 6.50 (s, 4H, 2 ×CH_2_), 8.11–8.14 (m, 2H, 2 × ArH), 8.46 (d, *J* = 6.8 Hz, 2H, 2 × ArH), 8.55 (d, *J*= 8.3 Hz, 2H, 2 × ArH), 8.72 (s, 2H, 2 × CH), 8.90 (d, *J*= 7.1 Hz, 2H, 2 × ArH), 9.41 (d, *J*= 7.1 Hz, 2H, 2 × ArH), 10.27 (s, 2H, 2 × ArH). ^13^C NMR (126 MHz, CD_3_OD-d_4_): *δ* 88.4, 126.0, 129.8, 131.5, 132.8, 133.3, 134.9, 137.1, 139.7, 148.0, 151.5. HRMS (HESI^+^): [M]^+^: calculated for C_22_H_20_N_4_O_3_^2+^ (*m/z*): 194.0762; found: 194.0763.

### Cholinesterase preparation

Human erythrocyte lysate containing AChE (3.1.1.7) and human plasma containing BChE (EC 3.1.1.8) were prepared at the Department of Toxicology and Military Pharmacy (University of Defence, Hradec Kralove, Czech Republic). The blood samples were collected from healthy volunteers from the vein into a disposable syringe containing 3.8% sodium citrate (the ration blood/citrate was 1:10 w/w). The citrated blood was centrifuged for 20 min at 2856 × g and the plasma was removed. The erythrocytes were washed three times with 100 mM phosphate buffer (pH 7.4) and then hemolysed in 100 mM phosphate buffer (pH 7.4) in a ratio 1:10 (w/w), frozen, and kept under −80 °C^11^. Human plasma was used as a source of BChE and was prepared from heparinised human blood. Blood was centrifuged for 20 min (4 °C, 5000 RPM) by Hettich Universal 320 R centrifuge. The plasma was separated and stored at −80 °C.

The recombinant form of human AChE/BChE was prepared in Department of Chemistry (Faculty of Science, University of Hradec Kralove, Hradec Kralove, Czech Republic)^12^ and purified using NGC Medium-Pressure Chromatography System (Bio-Rad, Hercules, CA). The total volume of 6–8 ml of medium containing secreted protein was desalted using 5 ml HiTrap Desalting column (GE Healthcare, Chicago, IL) equilibrated with buffer A (20 mM sodium phosphate buffer, 150 mM NaCl, 15 mM imidazole and 20% glycerol; pH 7.4). Acquired supernatant was loaded onto a 1 ml HisTrap FF column (GE Healthcare, Chicago, IL) equilibrated with buffer A. Captured proteins were eluted with buffer B (20 mM sodium phosphate buffer, 150 mM NaCl, 500 mM imidazole and 20% glycerol; pH 7.4). Imidazole was subsequently removed by repeated centrifugation in Amicon Ultra-4 (Ultracel-10K) tube (Merck Millipore, ‎Burlington, MA). Protein concentration was determined by linearised Bradford method adapted for 96-well plate.

### Inhibition assay

Inhibitory effect of the tested oximes on the AChE/BChE activity was determined using the Ellman’s method and is expressed as IC_50_ i.e. concentration that reduces the cholinesterase activity by 50%. 5,5′-dithiobis(2-nitrobenzoic acid) (Ellman’s reagent, DTNB), phosphate buffer (PB, pH 7.4), acetylthicholine (ATCh) and butyrylthiocholine (BTCh), were purchased from Merck, Prague, Czech Republic. For measuring purposes – polystyrene Nunc 96-well microplates with flat bottom shape (ThermoFisher Scientific, Waltham, MA) were utilised. All assays were carried out in 0.1 M KH_2_PO_4_/K_2_HPO_4_ buffer, pH 7.4. Enzyme solutions were prepared at 2.0 units/ml in 2 ml aliquots. The assay medium (100 µl) consisted of 40 µl of 0.1 M phosphate buffer (pH 7.4), 20 µl of 0.25 mM DTNB, 10 µl of the enzyme, and 20 µl of 0.25 mM substrate (ATCh iodide solution).

Inhibitor solutions in concentration range 10^−3^ – 10^−9 ^M were prepared. Tested compounds were 5 min preincubated. The reaction was started by the immediate addition of the substrate (20 µl). The activity was determined by measuring the increase in absorbance at 436 for AChE/412 nm for BChE at 37 °C at 2 min intervals – using a Multi-mode microplate reader Synergy 2 (Vermont, Perkinsville, VT). Each concentration was assayed in triplicate. Software GraphPad Prism version 5 (San Diego, CA) was used for the statistical data evaluation and IC_50_ values were calculated.

### Reactivation assay

Reactivation ability of oxime **30** was evaluated on human erythrocyte AChE and reactivation ability of oximes **17**–**18** and **30**–**32** was evaluated on human plasmatic BChE. Enzyme was inhibited by the solution of appropriate cholinesterase inhibitor – sarin, VX and paraoxon in propan-2-ol at concentration 10^−5 ^M for 60 min. Excess of OP inhibitor was subsequently removed using octadecylsilane-bonded silica gel SPE cartridge. The assay medium (100 µl) consisted of 40 µl of 0.1 M phosphate buffer (pH 7.4), 20 µl of 0.25 mM DTNB, 10 µl of the inhibited enzyme, 10 µl of oxime (concentration 100 µM or 10 µM) and it was incubated for 10 min at 37 °C. The reaction was started by addition of substrate ATCh/BTCh (0.25 mM, 20 µl). Activity of AChE/BChE was measured spectrophotometrically at 436 nm by the modified method according to Ellman^13^ using Multi-mode microplate reader Synergy version 2 (Vermont, Perkinsville, VT). Each concentration of reactivator was assayed in triplicate. The obtained data were used to compute reactivation potency (*R*; Equation (1). Results were corrected for oximolysis and intrinsic inhibition of by oxime reactivator.

(1) $$R=\left(1-\frac{\Delta A_{0}-\Delta A_{r}}{\Delta A_{0}-\Delta A_{i}}\right)\times 100\left[\%\right]$$

Δ*A*_0_ indicates absorbance change caused by intact AChE/BChE (phosphate buffer was used instead of AChE/BChE inhibitor solution), Δ*A*_i_ indicates absorbance change provided by cholinesterase exposed inhibitors and Δ*A*_r_ indicates absorbance change caused by AChE/BChE incubated with solution of reactivator.

### Reactivation kinetics

Selected compound (**17**) with best reactivation ability was further analysed in order to get reactivation kinetics parameters as follows. Human recombinant BChE was inhibited by 25 µM 4-nitrophenyl isopropyl methylphosphonate (NIMP-sarin surrogate) or 4-nitrophenyl ethyl methylphosphonate (NEMP-VX surrogate) for 30 min in order to obtain >99% inhibition. The excess of OP was removed by dialysis against 25 mM Na-phosphate buffer (pH 7.4) for 16 h (two buffer exchanges). Inhibited enzyme was incubated for 8 different times (0.5, 1, 1.5, 2, 5, 8, 10, and 15 min) with 7 different concentrations of tested oxime (5, 10, 30, 50, 80, 100, and 200 µM). The reaction mixture (100 µl final volume) consisted of 10 µl of inhibited enzyme (1.95 ng of total protein), 20 µl of 2.5 mM DTNB, 10 µl of corresponding oxime solution and 50 µl of 25 mM Na-phosphate buffer (pH 7.4). The reaction was started by addition of 10 µl of 10 mM substrate BTCh. The catalytic activity of reactivated enzyme was measured spectrophotometrically at 436 nm using Spark multimode microplate reader (Tecan, Männedorf, Switzerland). Acquired data were analysed by non-linear regression analysis according to Worek et al.^14–16^ using GraphPad Prism version 8.2 (San Diego, CA).

### Molecular docking procedure

From the online PDB database (rcsb.org), six various models of *h*AChE and *h*BChE (pdb id: 4ey7, 4bds, 5fpq, 6cqt, 5hf9, 2xqf) were downloaded and prepared for flexible molecular docking by MGL Tools utilities. The preparation of the receptors involved removal of the surplus copies of the enzyme chains, nonbonded inhibitors, addition of polar hydrogens, and merging of nonpolar ones. Default Gasteiger charges were assigned to all atoms. Flexible parts of the enzyme were determined by a spherical selection of residues (R = 11 Å) around the centre of the co-crystalised inhibitor in the enzyme active site. In the same points, the centres of the grid box of 33 × 33 × 33 Å were positioned. The rotatable bonds in the flexible residues were detected automatically by the AutoDock Tools version 1.5.4 programme (Scripps Research Institute, La Jolla, California, USA) . Given the limitation of the programme used for flexible molecular docking, water molecules had to be removed from the system. The flexible receptor parts contained 30–40 residues. The studied ligands were first drawn in HyperChem version 8.0 (Hypercube Inc., Gainesville, Florida, USA), then manually protonated as suggested by MarvinSketch version 6.2.0 software (http://www.chemaxon.com; ChemAxon Ltd., Budapest, Hungary), geometrically optimised by the semiempirical quantum-chemistry PM3 method, and stored as pdb files. The structures of the ligands were processed for docking in a way similar to the above-mentioned flexible parts of the receptor by AutoDock Tools version 1.5.4 programme. Molecular docking was carried out with the AutoDock Vina version 1.1.2 programme (Scripps Research Institute, La Jolla, California, USA) utilising computer resources of the Czech National Grid Infrastructure MetaCentrum. Each docking task was repeated 10 times with the exhaustiveness parameter set to 16, employing 16 CPU in parallel multithreading. From the obtained results, the solutions reaching the minimum predicted Gibbs binding energy were taken as the top-scoring modes. The graphic representations of the docked poses were rendered in PyMOL version 1.3 (the PyMOL Molecular Graphics System, version 1.5.0.4 Schrödinger, LLC New York, NY).

## Results and discussion

### Structural design and chemical synthesis

The isoquinolinium moiety represents structural template in the design of novel compounds. It is bulkier compared to pyridinium scaffold, ubiquitous feature in the commercial AChE reactivators. The isoquinolinium moiety was chosen for expected stronger binding *via* cation-π interactions with the cholinesterase binding sites which was previously confirmed for isoquinolinium AChE inhibitors^17^. Oxime moiety at position 5 of isoquinolinium scaffold was selected to plausibly effectively reactivate phosphylated AChE or BChE and, simultaneously, being able to penetrate to the close proximity of phosphylated serine within the active site. The peripheral binding ligands differ and include, for instance, bulky scaffolds like 5-carbaldoximequinolinium moiety attached *via* linker or medium size scaffolds like pyridinium amide attached *via* linker in order to define possible differences in further interactions with the AChE or BChE binding sites. Notably, pyridinium amides were formerly highlighted for binding within AChE peripheral site^18^.

**Scheme 1.**
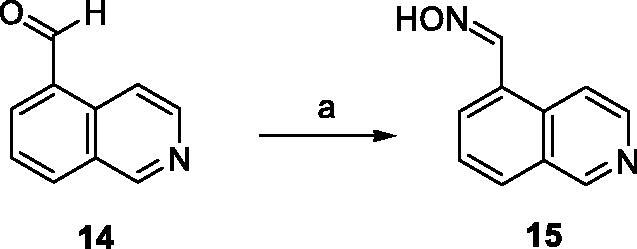
Preparation of aldoxime **15**. Reagents and conditions: (a) NH_2_OH, EtOH, 24 h, rt, 70%.

Regarding the synthesis, aldoxime precursor **15** was prepared by nucleophilic addition of a hydroxylamine (NH_2_OH) to the aldehyde **14** in EtOH in 70% yield (Scheme 1).

**Scheme 2.**
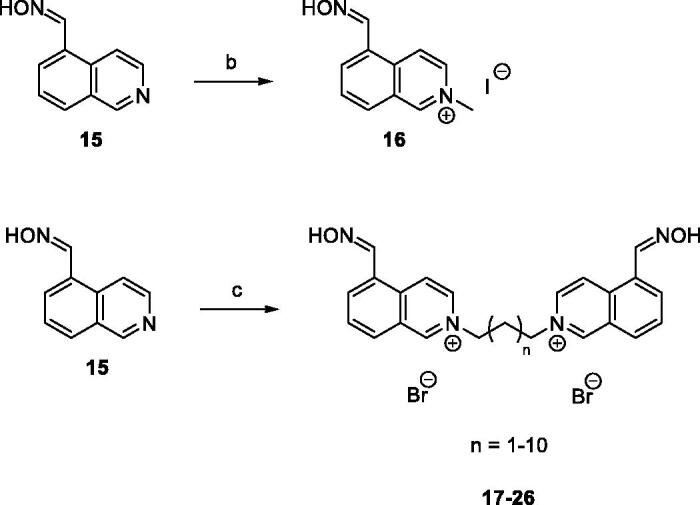
Preparation of quaternary salts **16**–**26**. Reagents and conditions: (b) CH_3_I, EtOH, reflux, 24 h, 71%; (c) dibromoalkanes, DMF, 73 °C, 48 h, 33–94%.

Subsequent nucleophilic bimolecular substitution (S_N_2) of **15** either with the methyl iodide (CH_3_I) or dibromoalkanes allowed formation of isoquinolinium salt **16** or bisisoquinolinium salts **17**–**26**, respectively, with intermediate to quantitative yields (Scheme 2).

**Scheme 3.**
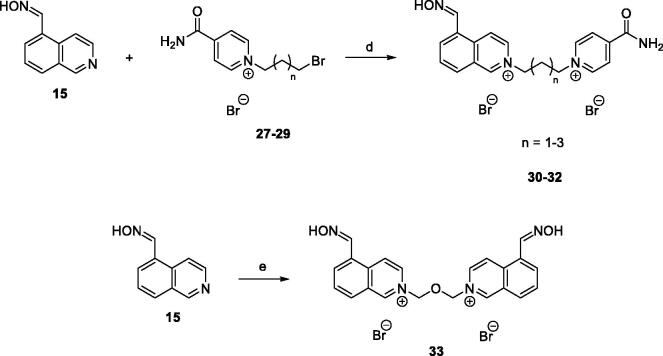
Preparation of bisquarternary salts **30**–**33**. Reagents and conditions: (d) DMF, 73 °C, 48 h, 53–77%; (e) bis(chloromethyl)ether, DMF, 73 °C, 48 h, 45%

Further, the group of unsymmetrical isoquinoline-isonicotinamide bisquaternary salts was synthesized by S_N_2 reaction of aldoxime **15** with monoquaternary isonicotinamide salts **27**–**29**. The reaction was carried out in DMF at 73 °C, and final products **30**–**32** were obtained in 53–77% yield (Scheme 3). Monoquaternary salts **27**–**29** were prepared according to the procedure described by Chennamaneni et al.^19^. In this work, symmetrical bisisoquinolinium salt containing dimethylene ether chain was also synthesized by reacting aldoxime **15** with bis(chloromethyl)ether in DMF as a solvent. The product **33** was obtained in 45% yield (Scheme 3). Based on HPLC with UV detection (*λ* = 254 nm), the non-calibrated purity of all products was ≥95%.

### *In vitro* inhibition

All developed compounds and standard obidoxime were initially tested for inhibition of *h*AChE or *h*BChE. The obidoxime resulted as poor AChE inhibitor (IC_50_^∼^197 µM) and insignificant BChE inhibitor (IC_50_^∼^5440 µM). The majority of novel compounds were found to inhibit *h*AChE in nanomolar or low micromolar range (Table 1). The exception was found for compound **30** that revealed inhibition in micromolar scale (IC_50_^∼^116 µM). As expected, the compounds with longer linkers (e.g. **23–26** with nine to twelve membered chains) were found powerful *h*AChE inhibitors. This phenomenon was previously described for similar quaternary isoquinolinium or quinolinium compounds and also correlates well with the topology of AChE cavity^20^.

**Table 1.** Inhibition of *h*AChE and *h*BChE by prepared compounds.

Compound	Linker	PAS ligand	IC_50_*h*AChE ± SEM (µM)	% of *h*AChE activity treated with oxime (100 µM)^a^	IC_50_*h*BChE ± SEM (µM)	% of *h*BChE activity treated with oxime (100 µM)^a^	Selectivity index *h*BChE/*h*AChE
obidoxime (**11**)	CH_2_OCH_2_	4-pyrid. oxime	197 ± 8	62	5440 ± 552	99	27.6
**16**	CH_3_	–	23.6 ± 6.8	20	94.0 ± 5.5	49	4.0
**17**	(CH_2_)_3_	5-Isoq. oxime	32.9 ± 3.7	34	284 ± 22	67	8.6
**18**	(CH_2_)_4_	5-Isoq. oxime	3.74 ± 0.03	11	122 ± 5	64	32.6
**19**	(CH_2_)_5_	5-Isoq. oxime	3.21 ± 0.40	6	77.7 ± 8.1	43	24.2
**20**	(CH_2_)_6_	5-Isoq. oxime	0.56 ± 0.05	5	31.4 ± 2.5	31	56.1
**21**	(CH_2_)_7_	5-Isoq. oxime	0.38 ± 0.03	5	3.94 ± 0.36	17	10.4
**22**	(CH_2_)_8_	5-Isoq. oxime	2.42 ± 0.03	9	3.52 ± 0.15	9	1.5
**23**	(CH_2_)_9_	5-Isoq. oxime	0.20 ± 0.01	1	1.73 ± 0.07	5	8.7
**24**	(CH_2_)_10_	5-Isoq. oxime	0.10 ± 0.001	1	1.34 ± 0.07	8	13.4
**25**	(CH_2_)_11_	5-Isoq. oxime	0.061 ± 0.005	0	1.08 ± 0.07	3	17.7
**26**	(CH_2_)_12_	5-Isoq. oxime	0.061 ± 0.005	0	1.18 ± 0.06	6	19.3
**30**	(CH_2_)_3_	4-pyrid. amide	116 ± 19	53	2475 ± 429	97	21.3
**31**	(CH_2_)_4_	4-pyrid. amide	55.7 ± 9.4	37	2697 ± 358	92	48.4
**32**	(CH_2_)_5_	4-pyrid. amide	14.2 ± 1.4	19	2141 ± 278	88	150.8
**33**	CH_2_OCH_2_	5-Isoq. oxime	30.3 ± 3.0	26	35.8 ± 2.6	34	1.2

^a^= (Absorbance of uninhibited enzyme + oxime (100 µM)/absorbance of uninhibited enzyme)*100.

Additionally, the majority of the compounds were found slightly less potent *h*BChE inhibitors with IC_50_ values at micromolar range. Besides these, five compounds displayed high micromolar inhibition (**17**–**18**) or low milimolar (**30**–**32**) inhibition. All of them bore shorter linkers (three to five-membered chains) that are probably not able to optimally interact with active site of *h*BChE. Compared to AChE, this discrepancy is obviously ascribed to different structural aspects between cholinesterases^21^. Interestingly, three of them were found in the subgroup containing pyridinium amide (**30**–**32**) that were also similarly ranked as weak inhibitors of *h*AChE^18^.

### *In vitro* reactivation

For the purpose of reactivation screening, only slightly potent inhibitors of AChE or BChE should be considered in order to avoid cholinesterase inhibition increment already caused by OPs. From this point of view, the maximal attainable plasmatic concentration for animal/human should be considered. It is presumed that attainable plasmatic concentration is maximally 100 µM after i.m. administration of a high dose of reactivator^22^. Thus, two concentrations (100 and 10 µM, maximal attainable concentration and one order of magnitude lower concentration) were chosen for the screening and the prepared compounds with IC_50_ over 100 µM for AChE or BChE and residual enzyme activity over 50% were selected for reactivation experiments (Table 1).

For AChE, only compound **30** was found weak inhibitor and was tested for reactivation of sarin, VX or paraoxon inhibited *h*AChE and compared to obidoxime (Table 2). The obidoxime showed reactivation for all the tested OPs with less ability to counteract phosphylated VX. The isoquinolinium carbaldoxime with pyridinium amide moiety **30** resulted with minimal reactivation in case of all tested OPs and was expelled from further kinetic experiments.

**Table 2.** Reactivation of *h*AChE inhibited by sarin, VX and paraoxon by selected compounds.

	Sarin-*h*AChE % reactivation ± SD		VX-*h*AChE % reactivation ± SD		Paraoxon-*h*AChE % reactivation ± SD	
Compound	100 µM	10 µM	100 µM	10 µM	100 µM	10 µM
obidoxime (**11**)	36.3 ± 0.3	11.0 ± 0.1	20.0 ± 0.2	6.37 ± 0.25	73.0 ± 0.8	34.1 ± 0.4
**30**	2.46 ± 0.2	1.23 ± 0.35	2.83 ± 0.16	0.76 ± 0.16	2.1 ± 0.4	0.9 ± 0.4

For BChE, compounds **17–18** (inhibition of *h*BChE over 100 µM) and **30–32** (inhibition of *h*BChE over 1000 µM) were selected and tested for reactivation potential against sarin, VX or paraoxon inhibited *h*BChE and compared to obidoxime (Table 3). The obidoxime was found to have some reactivation ability for sarin and VX, but markedly lower for paraoxon. Apparently, some novel compounds (**17**–**18**) showed markedly improved reactivation than obidoxime for sarin (**17**), VX (**17**–**18**) and paraoxon (**17**) when tested at 100 µM. This finding seems to be important since obidoxime formerly resulted as the best reactivator of *h*BChE inhibited by tabun^23^, although its reactivation was found not appropriate for constructing a pseudo-catalytic scavenger. More interestingly, these symmetrical isoquinolinium carbaldoximes with three or four-member linkers were found better *h*BChE reactivators than obidoxime at 10 µM for sarin and VX. On the other hand, the isoquinolinium carbaldoximes with pyridinium amide moiety (**30**–**32**) which were poor BChE inhibitors were endowed with minimal reactivation for all tested OPs.

**Table 3.** Reactivation of *h*BChE inhibited by sarin, VX and paraoxon by selected compounds.

	Sarin-*h*BChE% reactivation ± SD		VX-*h*BChE% reactivation ± SD		Paraoxon-*h*BChE% reactivation ± SD	
Compound	100 µM	10 µM	100 µM	10 µM	100 µM	10 µM
obidoxime (**11**)	23.0 ± 0.4	6.4 ± 0.2	22.3 ± 0.5	5.0 ± 0.4	7.7 ± 0.9	2.0 ± 0.5
**17**	52.6 ± 1.3	14.7 ± 0.4	45.2 ± 0.5	14.3 ± 0.4	10.6 ± 0.5	2.0 ± 0.5
**18**	17.3 ± 0.8	4.8 ± 0.8	39.8 ± 0.4	11.5 ± 0.5	3.6 ± 1.0	0.2 ± 0.3
**30**	7.2 ± 0.5	4.8 ± 0.2	11.5 ± 1.1	2.0 ± 0.3	2.7 ± 0.5	2.0 ± 0.6
**31**	5.9 ± 0.3	4.2 ± 0.5	6.3 ± 0.6	0	1.1 ± 0.2	0
**32**	5.3 ± 0.4	3.9 ± 0.5	11.2 ± 0.4	2.8 ± 0.4	1.9 ± 0.3	1.9 ± 0.2

### Reactivation kinetics

The best reactivator of OP-inhibited *h*BChE was selected for further kinetic experiments. The affinity of oxime **17** towards OP-inhibited human recombinant BChE (reflected by *K*_D_) and the ability to remove the phosphyl residue from the active site of the enzyme (reflected by the reactivity constant *k*_r_) were determined (Table 4). The specific overall second-order reactivation rate constants (*k*_r2_=*k*_r_/*K*_D_) were calculated. Although compound **17** showed a significantly higher affinity for the phosphylated enzyme, its ability to remove OP-moiety from hBChE was found lower compared to obidoxime for both used OPs. However, the overall reactivation rate indicated increased reactivation of NEMP (VX surrogate)-inhibited BChE by compound **17**, when it resulted slightly lower for NIMP (sarin surrogate)-inhibited BChE if compared to obidoxime.

**Table 4.** Reactivation kinetics of human recombinant BChE inhibited by NIMP (sarin surrogate) and NEMP (VX surrogate) using selected compounds.

	NIMP (sarin)			NEMP (VX)		
Compound	*K*_D_ [µM]	*k*_r_ [min^−1^]	*k*_r2_ [mM^−1^min^−1^]	*K*_D_ [µM]	*k*_r_ [min^−1^]	*k*_r2_ [mM^−1^min^−1^]
obidoxime (**11**)	188.7	6.71	35.5	86.5	5.08	58.7
**17**	17.4	0.47	27.1	5.3	1.29	244.5

### Molecular docking studies

In order to interpret obtained *in vitro* results, the molecular docking was conducted. Prediction of binding modes and binding affinities of the studied compounds in *h*AChE, *h*BChE, sarin-*h*AChE, VX-*h*AChE, POX-*h*AChE, and VX-*h*BChE models was performed with imposing torsional flexibility on several tens of the active site residues in AutoDock Vina version 1.1.2 software, employing high-performance computing grid network^24^.

**Figure 3.**
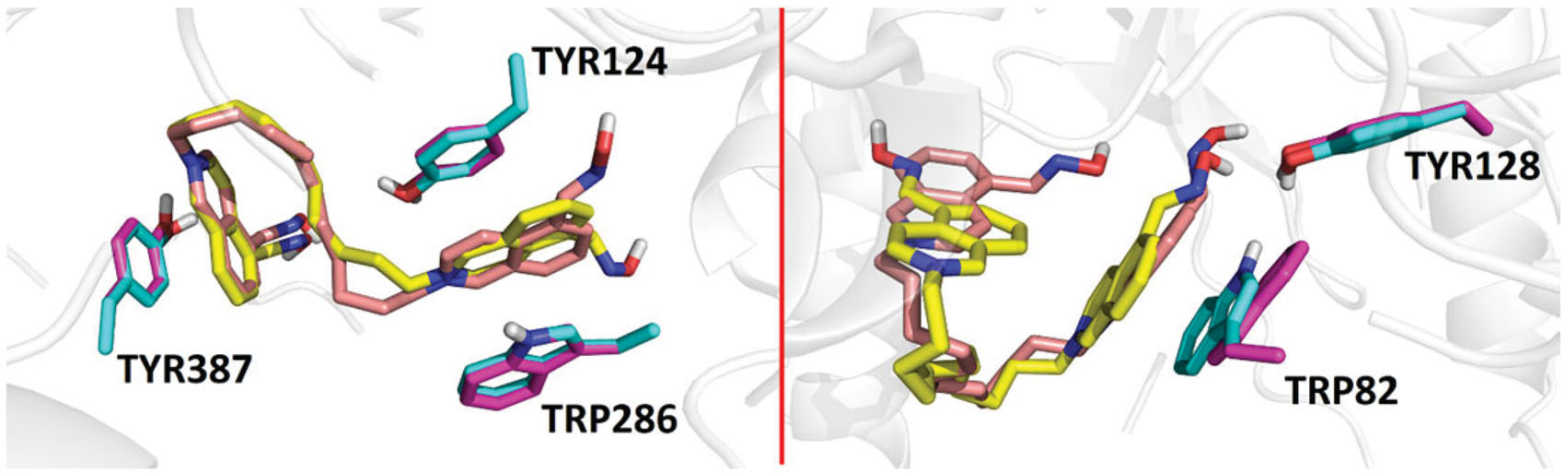
Overlaid predicted binding modes of compounds **25** (yellow) and **26** (light pink) in *h*AChE (pdb id: 4ey7, left) and *h*BChE (pdb id: 4bds, right). The residues interacting with **25** and **26** are coloured in light green and purple, respectively.

From the binding energy estimates of the top-scoring binding modes of the studied compounds and obidoxime in *h*AChE (pdb id: 4ey7), it seems probable that lengthening of the linker in bis-isoquinolinium aldoximes increases the binding affinity, which is also in a good correlation with decreasing experimental values of pIC_50_ for *h*AChE (*R*^2^=0.67, *p*= .0025). The strongest inhibition of *h*AChE *in vitro* was observed for compounds **25** and **26** that provided binding energy estimate of −13.9 and –13.7 kcal/mol, respectively (Table 5). A very similar trend for the IC_50_ values and the number of carbon atoms in the linker of bis-isoquinolinium aldoximes can be seen in the case of *h*BChE inhibition. Again, ligands **25** and **26**, containing 11 and 12 carbons in the linker chain, showed the strongest experimental inhibition potency in *h*BChE. On the other hand, changes in the binding energy estimates of bis-isoquinolinium aldoximes and obidoxime in *h*BChE (pdb id: 4bds) correlate significantly worse with the values of pIC_50_ than it is observed in the case of *h*AChE inhibition (*R*^2^=0.35, *p*= 0.0176). In *h*BChE, compounds **25** and **26** provided binding energy estimates of –11.1 and –11.0 kcal/mol. In the case of *h*AChE simulation, compounds **25** and **26** occupy approximately the same region in the enzyme active site in which the compounds are stabilised by π–π stacking with TYR124, TRP286 and TYR337 (Figure 3, left). These interactions are considerably limited in *h*BChE model, where the compounds **25** and **26** are fixed in the binding mode by π–π stacking only with TRP82 and by hydrogen bonding with TYR128 (Figure 3, right).

**Figure 4.**
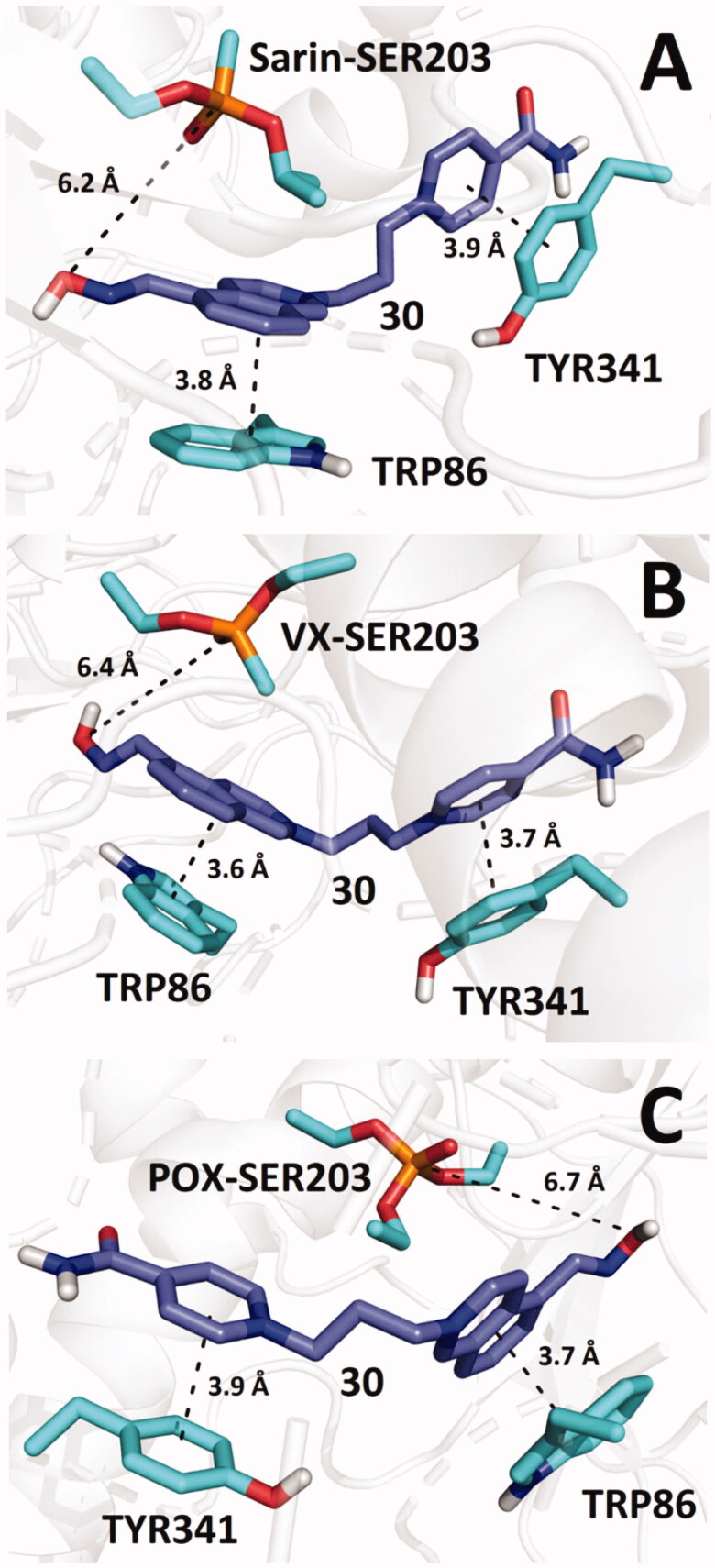
Predicted binding mode of compound **30** (blue) in *h*AChE inhibited by sarin (**A**; pdb id: 5fpq), VX (**B**; pdb id: 6cqt) and paraoxon (**C**; pdb id: 5hf9).

Further molecular docking studies with *h*AChE inhibited by sarin, VX and POX revealed that the predicted binding energy of bis-isoquinolinium aldoximes reaches the optimal values if the linker contains 5 or 7 carbon atoms (i.e. **19** and **21**). Nearly in all three cases of OP-inhibited *h*AChE, the estimated binding energy for bis-isoquinolinium aldoximes are higher than the estimated binding energy resulting from molecular docking of the compounds in intact *h*AChE model. Only the bis-isoquinolinium aldoxime **30** exhibited some reactivation potency for all types of OP-inhibited *h*AChE, whilst its estimated binding affinity in the inhibited enzymes reached relatively low levels (Figure 4; i.e. sarin-*h*AChE –12.1 kcal/mol, VX-*h*AChE –11.6 kcal/mol, POX-*h*AChE –11.9 kcal/mol). Other isoquinolinium aldoximes exhibited mostly stronger *in silico* binding energy for OP-inhibited *h*AChEs. It can thus be hypothesized that high binding energy of the bis-isoquinolinium aldoximes decreases the reactivation ability. This may be a result of stronger inhibition ability, which is supported by stronger binding of the reactivator in the OP-inhibited active site.

Compound **30** interacts with TRP86 and TYR341 by π–π stacking in such a way that the aldoxime group is not optimally pointed towards the phosphorus atom in OPs. Nonetheless, similar spatial arrangement of aldoxime reactivators can be found in various docking studies and even in X-ray models of OP-inhibited AChE, co-crystalised with aldoximes (e.g. HI-6 in model pdb id: 5fpq)^25^^,^^26^. Despite the fact that molecular mechanics cannot in principle predict the necessary orientation of the aldoxime group towards the phosphorus atom because this follows only from quantum chemistry calculations, such initial unfavourable geometric arrangements suggest that reactivation requires certain degree of conformational flexibility of the moiety bearing the aldoxime function to enable reactivation. High flexibility of the pyridinium aldoxime moiety was observed in computational simulations and confirmed in X-ray experiments for example in the case of sarin-inhibited *h*AChE reactivation by HI-6^25^.

An opposite relationship was found when the predicted binding energies were compared with reactivation of *h*BChE inhibited by VX. The best reactivation ability *in vitro* was observed for compound **17** which also exhibited the highest binding energy estimate in *h*BChE-VX model (−12.3 kcal/mol). Comparing both trends in OP-*h*AChE/*h*BChE, it can be hypothesized that the binding energy of the reactivator in the inhibited enzyme active site has to reach an optimal value (i.e. approximately around −12.0 kcal/mol), otherwise, it loses its reactivation ability. Apparently, bis-isoquinolinium aldoximes exhibiting *in silico* binding energy far from the supposed optimum proved practically no reactivation potency in the studied OP-*h*AChE/*h*BChE complexes. According to several quantum-chemical simulations of similar reactivations processes in OP-*h*AChE/*h*BChE, it seems that binding of the aldoxime reactivators in the enzymes’ active sites stabilises the transitions states in the reactivation coordinate^27^. Intermolecular interactions of aldoxime reactivators with residues in *h*AChE/*h*BChE influences also deprotonation of the oxime function of positively charged reactivators and contributes to release of serine anion and to its neutralisation^28^. Nonetheless, more accurate studies, involving explicit water molecules, have to be performed in order to elucidate the relationships between the binding strength and the reactivation potency^29^.

**Figure 5.**
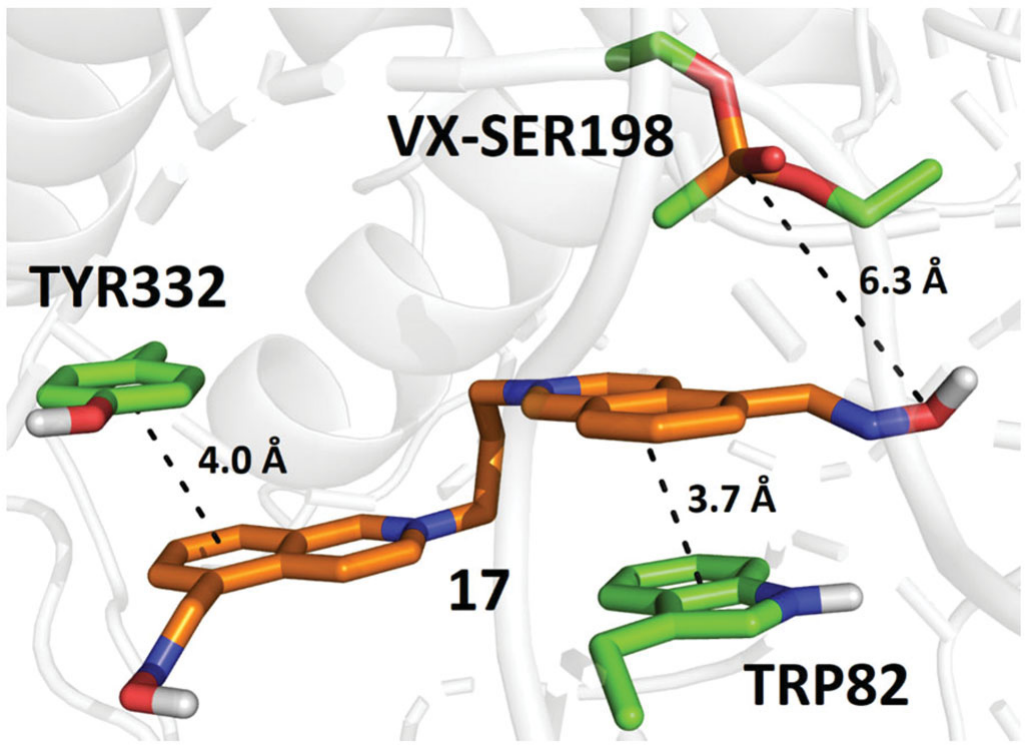
Predicted binding mode of compound **17** (orange) in *h*BChE inhibited by VX (pdb id: 2xqf).

The binding mode of **17** in *h*BChE-VX predicted by molecular docking, which exhibited the highest reactivation potency, is shown in Figure 5. Compound **17** interacts with the enzyme mainly through π–π stacking with TRP82 and TYR332. The predicted binding energies of the studied compounds in the selected cholinesterase models are summarised in Table 5.

**Table 5.** The lowest predicted binding energies of the studied compounds in selected cholinesterase models by molecular docking.

Compound	Binding energy estimate [kcal/mol]					
	*h*AChE	*h*BChE	*h*AChE-VX	*h*AChE-SAR	*h*AChE-POX	*h*BChE-VX
**HI-6**	−9.6	−8.6	−9.9	−13.5	−14.7	−7.9
Obidoxime (**11**)	−9.8	−8.1	−9.6	−10.0	−9.9	−9.2
**16**	−9.3	−7.6	−9.6	−9.3	−9.2	−8.3
**17**	−12.5	−11.7	−12.9	−14.0	−13.2	−12.3
**18**	−12.0	−11.3	−13.5	−13.5	−13.4	−11.7
**19**	−12.2	−11.1	−14.1	−14.2	−14.7	−10.8
**20**	−12.6	−11.1	−13.6	−14.5	−14.2	−10.0
**21**	−12.6	−11.3	−13.7	−14.9	−13.4	−10.7
**22**	−12.6	−10.8	−13.6	−14.7	−13.2	−11.0
**23**	−13.4	−11.1	−13.7	−14.8	−13.0	−10.1
**24**	−13.6	−11.4	−12.8	−14.7	−12.9	−9.9
**25**	−13.9	−11.1	−13.5	−12.7	−12.9	−10.7
**26**	−13.7	−11.0	−12.8	−12.9	−12.7	−10.0
**30**	−11.0	−10.4	−11.6	−12.1	−11.9	−11.0
**31**	−11.3	−10.4	−11.3	−11.9	−11.9	−10.2
**32**	−11.2	−9.5	−11.3	−12.0	−11.9	−10.6
**33**	−12.6	−11.5	−12.6	−13.8	−9.2	−11.7

## Conclusion

In summary, the series of fifteen symmetrical and unsymmetrical isoquinolinium-5-carbaldoximes was designed and prepared. The novel compounds were evaluated for intrinsic *h*AChE and *h*BChE inhibition, when the majority of novel compounds resulted with high inhibition of both enzymes. The selected compounds with lower inhibitory ability were further tested for reactivation of *h*AChE or *h*BChE inhibited by sarin, VX or paraoxon. The AChE reactivation for all used OPs was found negligible if compared to the reactivation of obidoxime. On the contrary, two compounds were found to reactivate *h*BChE inhibited by sarin or VX in better way than obidoxime at human attainable concentration. One compound resulted as better reactivator to obidoxime for NEMP (VX-surrogate)-inhibited BChE. The *in vitro* results were further compared to *in silico* data determined by molecular docking studies and rationalized. The obtained data possess important structural insight and future directions on designing potent BChE reactivators.
